# Perforation of the Stomach, without Ulceration or Softening of Its Coats

**Published:** 1830-03

**Authors:** Leonard Peirce

**Affiliations:** Sutton, Massachusetts.


PERFORATION OF THE STOMACH.

*Perforation of the Stomach, without Ulceration or Softening of its Coats.* By LEONARD PEIRCE, M.D. of Sutton, Massachusetts.

FLETCHER BOTTOMLY, a native of Cheshire, England, came to the United States in June 1827, then aged nineteen years; he followed weaving in a woollen manufactory.

January 30th, 1829.—I was called about six o'clock this evening to visit him, but, being from home, did not see him till nearly eight. I found him suffering from severe pain in the region of his stomach; feet and hands cold; pulse small and fluttering; countenance contracted and anxious. On inquiring of himself and his comrades, I learned that he had been as well as usual till about five this evening, when he was suddenly seized with a violent pain at the epigastrium, which soon extended downwards, but the seat of the pain remained at the stomach. When first seized, the pain was so violent that he cried out "I am dying," and threw himself upon the floor, holding his bowels with his hands, and pressing his body and thighs together. He was soon helped to his lodgings, which were a few rods distant, but was unable to walk upright, remaining bent, supporting himself with his hands upon his knees. The pain still continued violent, but was not now confined to his stomach, being occasionally as low down as the pubic region. Before I arrived, he had taken an emetic of ipecacuanha, containing eight grains of calomel, which had vomited him twice with some relief; but the pain being now in his stomach, I gave him tepid water, which vomited him twice more, and he expressed himself considerably relieved. I now applied flannels wet with warm water to his extremities, and gave him two grains of solid opium. His extremities soon became warm; pulse fuller and stronger; the pain abated considerably, and he fell asleep. I now directed one ounce of castor oil to be given every three hours until his bowels were moved, and left him for the night.

31st.—At seven A.M. I found him considerably prostrated, and in rather severe pain; pulse fluttering, and extremities cold. The bowels were fuller than natural, but were not tender on pressure. Had passed a restless night, and taken the oil without producing any sensation of motion in his bowels; much thirst. I now divided two drops of croton oil into six parts, and directed one part to be given every half-hour until the whole was taken, unless a motion was produced. Stimulants were given, and water-gruel for drink.

Four P.M.—Had taken all the croton oil, without producing the slightest cathartic effect. Pulse much as in the morning. Complained of considerable soreness in his bowels, which were rather fuller than in the morning. I should have given enemata, but, for want of the proper apparatus, was obliged to postpone them, and directed one ounce of castor oil to be given every hour, and put a blister upon the epigastrium.

Eight P.M.—Directed an enema of decoction of Senna, which passed off in about twenty minutes, without bringing any fæces with it. In a few minutes I repeated the injection, which soon passed off unmixed with any alvine matter. I directed an enema of milk and molasses, of each four ounces, to be given every hour until his bowels were moved; continued the drink of water-gruel; left him for the night.

February 1st, four A.M.—Vomited a small quantity of dark, fetid liquor, and in about fifteen minutes expired.

I very readily obtained leave of his friends to examine the body; which I did at two P.M., ten hours after his death.

The blister had produced very slight vesication, and the bowels were considerably tumid. On cutting through the parietes of the abdomen, there was a sudden gush of liquor, consisting of those articles he had taken into his stomach, castor oil, water-gruel, &c. I observed to the bystanders that there was a rupture of the stomach or intestines; and I then supposed it to be from ulceration. After removing the fluids from the abdominal cavity with an injecting syringe, I laid open the abdomen, and proceeded to search for disease. The vessels of the omentum, and of the peritoneal coat of the intestines, were considerably gorged with blood; but there were no unnatural adhesions between any of the parts. The mucous coat of the intestines was of a healthy appearance, except in some places in the small intestines there were minute scarlet dots thickly set together. The urinary bladder was entirely empty, and of a healthy appearance. Kidneys healthy. The liver was of a pale ash-colour externally, and internally much paler than natural. On arriving at the stomach, I found, about half an inch above the pylorus, on the anterior part, an opening about two and a half lines in diameter. This had the appearance of being punched out with a cutting instrument, and was not much unlike the holes made in harness for the buckle tongues; but the edges were not quite so well defined as though cut with an edged tool. There was no appearance of disease about the perforation, either externally or internally, except that the mucous lining of the stomach was filled with black and brown dots, of about the same appearance, except in colour, as the grains of Indian meal taken in gruel.

Bottomly was of a melancholic temperament, tall, spare, and temperate in the use of spirituous liquors He was a voracious eater, devouring as much at his regular meals as two common eaters, and frequently eating between meals, and always taking some cold food just before going to bed. He had, for several years previous to his death, been afflicted with purulent ophthalmia. Since the time of his arrival in this country, he had been very costive, generally not having a stool oftener than once a week. He had, within three or four months of his death, three small, hard, red, or rather purplish tumors, directly in the pit of his stomach, which were very sore and painful, slow in forming, and difficult to cure. He usually applied a plaster of shoemaker's wax to them, which caused them to ulcerate, and to discharge a sanious, bloody matter ; and then they would heal. The last one was a little previous to his death, but had at the time got entirely well.*

---

### TURPENTINE IN HERNIA.

* American Journal of Med. Sciences.

